# Influence of culture on dietary practices of children under five years among Maasai pastoralists in Kajiado, Kenya

**DOI:** 10.1186/s12966-015-0284-3

**Published:** 2015-10-08

**Authors:** Peter M. Chege, Judith O. Kimiywe, Zipporah W. Ndungu

**Affiliations:** Department of Food, Nutrition and Dietetics, Kenyatta University, Thika Road, Nairobi, Kenya; Department of Nutrition and Dietetics, Mount Kenya University, General Kago Road, Thika, Kenya

**Keywords:** Culture, Dietary practices, Undernutrition, Pastoralist, Maasai

## Abstract

**Background and objectives:**

Globally, children aged under five years are prone to malnutrition. Maasai are a nomadic community in Kenya still upholding traditional and has a high rate of child undernutrition. Consideration of cultural practices is a pre-condition for ensuring appropriate dietary practices. However, information on the influence of culture on dietary practices among Maasai children is minimal. The possible influence of culture on dietary practices among these children was investigated.

**Methods:**

Six focus group discussions sessions each consisting of 10 mothers were conducted from two randomly selected villages in Sajiloni location, Kajiado County.

**Results:**

Results from this study showed that children mainly consume cereals and legumes. Nomadism makes animal products inaccessible to most children. Livestock are considered a sign of wealth, thus mainly slaughtered on special occasions. Additionally, selling of animals or animal products is not encouraged limiting income that would improve the food basket. Some food taboos prohibit consumption of wild animals, chicken and fish limits the household food diversity. Consumption of vegetables is limited since they are perceived to be livestock feed. The belief that land is only for grazing contributes to low crop production and consumption thus the diets lack diversification. Maasai culture encourages introduction of blood, animal’s milk and bitter herbs to infants below six months, which affects exclusive breast feeding. The men are prioritized in food serving leading to less and poor quality food to children. The consumption of raw meat, milk and blood is likely to lead to infections. The practice of milk fermentation improves bioavailability of micronutrients and food safety. Socialism ensures sharing of available food while believe in traditional medicine hinder visit to health facilities thus no access to nutrition education.

**Conclusion:**

This study concludes that culture influence the dietary practices among children under five years. It recommended initiation of programs to create awareness on how the beliefs negatively affect dietary practices with a view for a change.

## Background information

Globally, cultural practices are vital in communities and are known to influence dietary practices [6, 11, 32, 34]. Children under five are the most vulnerable groups to nutrient deficiencies. These deficiencies are even worse in arid and semi-arid lands (ASAL) due to food insecurity. In Kenya, food insecurity is common in ASAL counties and among pastoral communities.

There is high infant mortality rate among pastoralists in ASAL [19]. Kajiado County is one of the ASAL Counties in Kenya, mainly dominated by the Maasai community. It has a population of 406,000 people [3] with seven administrative divisions, Central, Isinya, Loitokitok, Magadi, Mashuru, Namanga and Ngong [16]. Only 7 % of its range land is potential for agriculture. The largest portion of the County is characterized by poverty, poor infrastructure, insecurity, frequent droughts and limited livelihood options [3]. Most of the agricultural potential areas is in the Athi-Kapiti Plains and in the south of the District, along the Kilimanjaro foothills. Mean annual rainfall ranges from 300 to 800 mm and is mainly bimodal, with short rains from October to December and long rains from March to May [4]. Despite the high development potential, the ASALs have the lowest development indicators and the highest poverty incidence amongst all areas in Kenya [19].

Maasai speak *Kimaasai* language and they are well known for their distinctive customs [7]. They are nomadic pastoralists and their life revolves around the herds of cattle [33]. According to tradition, Maasai believe that all cattle belong to them and a sign of wealth, Cattle traditionally are used to pay dowry. According to Lauren et al. [23], only men have rights to the cattle and women are wholly dependent. The community has a polygamous structure characterized with many children.

The Maasai families live in an enclosed homestead known as a *Manyatta* with a fence made using thorny bushes surrounding the *Manyatta* to protect them and their livestock from intruders and predators. Each *Manyatta* has about 10 to 20 huts known as "*Inkajijik*" or “*boma”* [9]. However, in the recent past the average size of the *boma* has declined and the single family unit has become increasingly common as the Maasai became increasingly sedentary and as they move towards individualization in terms of land ownership [26]. Men and women have distinct roles. Women are responsible for construction of houses, collecting firewood, fetching water, milking the herds of cattle and cooking for the family whereas the young boys look after the livestock while the warriors maintain security. Older men manage daily operations in the community [23]. Typically in Maasai culture, men are the sole decision makers. The limited access to financial resources and decision making among women has direct implications on the health of the women and their children [7].

Traditionally, the main livelihood among Maasai pastoralists was animal production [5]. The Maasai traditional diet comprises of raw blood, milk, fat, honey, meat and tree bark [27].

With civilization and the influence of the western culture, strong Maasai beliefs are partly changing. However, a significant of Maasai community still upholds to some cultural identities. Due to climate change and the need to adapt, the Kenyan government has instituted programs towards behaviour change from the traditional semi-nomadic lifestyles. This has not been fully realized because majority has continued with the age-old customs.

Traditionally, the dietary practices among the Maasai were appropriate, except for the practice of exclusive breastfeeding and intake of raw food. These foods could meet the nutrients required for a healthy living. However, there has been an ongoing nutrition transitions among pastoralists due to western influence and decline in animal production due to climate change. Thus, some traditional cultures need to be addressed to match these changes occurring in the developing countries as a result of globalization. However, this cannot be achieved with the scarcity of information on how the cultures affect the dietary practices for children under five years.

There still exist challenges in reducing the rates of under nutrition, with one of the contributing factor being poor dietary practices [14]. A study by Morrell [25] and Johnson et al. [18] found a relationship between culture and dietary habits. Other studies have shown cultural beliefs to be an indicator of what people value as important diets, less valuable diets as well as diets that should not be consumed (Kittler, Sucher and Nelms, [21, 38]). Studies conducted by Lyana and Manimbulu [24] and Trefry, Parkins and Cundill [35], highlights that culture influence the diets adopted and consequently the food security status of households.

Since Maasai people still strongly holds the cultural practices, there are information gaps on how their culture affects dietary practices. There is thus need to explore the effect of the cultural beliefs on the dietary practices of children under five years. It’s in this view that the study examined the influence of cultural aspect on dietary practices of children under five years in Kajiado County.

## Methods

The study used both qualitative methods to explore the effect of the Maasai culture on the dietary practices of the children under five years. Kajiado County, Kajiado South district, Central division were purposively selected as they are predominantly occupied by Maasai community with strong cultural beliefs which they still uphold to date. One location; (Sajiloni location) and one sub-location (Sajiloni sub-location) were randomly selected. Two out of eight villages in Sajiloni sub-location were randomly selected using random number generator. These were Sajiloni and Nameyana. The eight villages are similar in terms of infrastructure, climate and cultural characteristics. Purposive sampling was used to select a homogenous group of mothers for each FGD.

Focus group discussions were used to reveal the opinions of the mothers about cultural orientation that still affect dietary practices among the children. To capture as much information as possible and get an in-depth perspective of the culture and dietary practices, a total of six focus groups from two randomly selected villages in Sajiloni Sub-location participated in this study. It is recommended that when only FGDs are used for data collection, at least four FGDs can be conducted. This study focused on six FGDs, to capture information from across all the various occupations and education categories [20, 31]. Each FGD consisted of 10 women with children under five.

The research permit was sought from the National Council for Science and Technology (Research Permit No. NCST/RRI/12/1/MED/236/5). Ethical clearance was obtained from Ethical Review Committee from Kenya Medical Research Institute (KEMRI) (Research Permit No. KEMRI/RES/7/3/1/267). An informed and signed consent was sought from respondents before the study. The research purpose and protocols were explained in detail to the local administration, community leaders and the respondents.

Focus group discussion (FGD) guides were used to collect data. Focus group discussions are excellent method for collecting qualitative data where participants are able to build upon one another’s comments, stimulate thinking and discussion, and thus generate ideas and breadth of discussion [22]. The interactive element makes focus groups ideally suited to explore issues, feeling and perceptions.

A primary school which was geographically convenient for the participants was selected. The participants were asked about the cultures that they still hold to, why the culture was valued and which particular ones affected the dietary practices of children. Interviews were mostly conducted in both Kiswahili and in *Kimaasai*. The session was audio-taped and notes were taken. Each FGD took about 60 min.

Interviewers developed transcripts from the interviews. Since the recordings were in Kiswahilii language and the notes in English, the preparation of the transcript involved a combination of translation and transcription of each FGD. For analysis of variables the data was entered into Excel sheets. For the qualitative analyses, the transcripts provided the data for text analysis. In the text analysis, the relatively small size of the database (*n =* 6 FGDS) made it unnecessary to use a software program to code variables. It was feasible to work directly with transcripts, creating files of statements on specific topics per each FGD. The data was then sorted and assigned to categories for coding. Thematic analysis of the notes and audio-tape transcripts was conducted. A verbatim transcript of each discussion was generated. The complete transcript was compared with the handwritten notes to fill any the gap. Analysis involved describing the data and interpreting the emerging themes.

## Results and discussions

### Demographic and socio-economic characteristics of the mothers

#### The consent rate was 100 %

The ages of the mothers ranged from 16 to 44 years. Most of the mothers, (33.3 %) were between the ages of 30–34 years. It was observed that more than 81 % of the mothers were below the age of 34 years. According to the Maasai culture, women get married at at an early age since most of them do not go beyond primary school education.

Majority (93.3 %) of the mothers were married (Table 1). Due to a culture that discourages divorce and promotes wife inheritance in case of death, there were few cases (<7 %) of mothers who were single, separated, divorced or widowed. The presence of a father in Maasai community is highly valued as women may not adequately provide for the children due to lack of resources.

**Table 1:** Socio-demographic and economic profile of the mothers of under five children in Kajiado County

Variable		Experimental (*N =* 60)	
Age of the mothers	16-20	3	5.0
	21-24	8	13.3
	25-29	18	30.0
	30-34	20	33.3
	35-39	9	15.0
	40-44	2	3.3
	Total	60	100.0
Marital status	Married	56	93.3
	Separated	1	1.7
	Single	2	3.3
	Widowed	1	1.7
	Total	60	100.0
Education level	Primary incomplete	30	50.0
	Primary complete	20	33.3
	Secondary	7	11.7
	Tertiary	3	5.0
	Total	60	100.0
Occupation	Herding	28	46.7
	Business/petty trade	11	18.3
	Formal employment	3	5.0
	Casual labourer	7	11.7
	Housewives	10	16.7
	Farming	1	1.7
	Total	60	100.0

*Majority of the mothers were mothers, between the age of 25–29 years and were married

Most of the respondents (50.0 %), had not completed primary education (Table 1). Traditionally the Maasai culture, girl child education is not emphasized and this could have contributed to the high number of women who with low education. Most of the mothers were involved in herding cattle.

Majority of the households had five members (33.3 %, Table 2). The minimum household size was 3 while maximum was 9. The mean household size was 5.2 ± 1.54 SD. This figure was higher than the national figure of 4.5 [19]. Giving birth to many children in the Maasai community is culturally encouraged leading to the large household sizes [10].

**Table 2:** Household monthly income among mothers of under five children and housed size in Kajiado County

		(*N =* 60)	
		*n*	%
Household income	<2000	5	8.3
	2001- 4000	12	20.0
	4001- 6000	23	38.3
	6001- 8000	16	26.7
	8001- 10000	3	5.0
	>10001	1	1.7
	Total	60	100.0
Household size	>8	6	10.0
	7	5	8.3
	6	11	18.3
	5	20	33.3
	4	11	18.3
	3	7	11.7
	Total	60	100.0

**Table 3:** Emerging themes

Practice	Outcome
Nomadism makes Animals to be away from home	Immediate animal products not available to children
Livestock is a sign of wealth thus Animals rarely slaughtered or sold	Lack of adequate food and income to buy food
Forbidden foods thus some available foods not being consumed	Lack of diversified diet
Land to be used for grazing leading to minimal crop production	Lack of adequate food
Early introduction of foods to infant below six months	Exclusive breast feeding affected
Poor intra-household food distribution	Men get more food while children get less
Consumption of raw animal products	May lead to infections
Milk fermentation	Improved bio-availability of micronutrients
Food sharing	The amount consumed is reduced for those who have and increased for those without
Reliance on traditional healersfor medication	Lack of access to adequate health care services

Almost all the households earned some income (Table 2). Majority of the households (38.3 %) earned between 4001–6000 KES. The average income per household was 5391.3 ± 1520 SD. The household incomes was in the lower range, with more than half earning less than Ksh 6,000 ($74 dollars) a month. Household income is a contributing factor to the dietary practices of a child [17]. Income has been shown to affect the ability to procure food [2, 28, 37]. Low income amidst high food costs are a barrier to the adoption of nutrient-dense diets [13].

### Dietary practices among the children

The respondents reported that all children were mainly being fed on thin or stiff porridge made from maize flour, rice and beans. Milk intake was minimal and mainly mixed with water, sugar and tea leaves with adequate consumption only when the animals had not moved. This resulted in children mainly being fed on cereals and legumes. The intake of fruits and vegetables was very low. From the discussions, the mothers reported that the number of food groups consumed per day was 3 which was low as Gibson and Hotz, [15] recommends consumption of more than four food groups per day.

This study notes that the low level of education among the mothers was a barrier to good dietary practices. The fact that most of the women had low education levels is an indicator of adoption of poor dietary practices, which is in line with a study by Abuya, Ciera, & Kimani-Murage [1]. A study by Doan [12] showed that education highly correlates with food security. According to KDHS [19], children of mothers with secondary or higher education are likely to have better nutritional status due to better child care practices compared to those who only attained up to primary education or with no education at all.

The children were mainly fed on three meals per day. This was below the recommended since children under five years are supposed to consume three main meals per day with snacks in between [30].

### Cultural practices and their effect on dietary practices

The various cultural practices were highlighted (Table 3). The results in this section focus on cultural practices that were noted to affect the dietary practices of children under five years.

This study noted that in Maasai culture men normally move with the livestock in search for pasture leaving women and children behind. This takes two weeks to three months limiting the children adequate access to the immediate animal products like milk and blood. With the change in climatic conditions, it was reported that the duration of men staying away from homes in search for pasture had prolonged. This leaves women to struggle while providing food for the family on their own.

It was noted that animals are highly valued as a sign of wealth and are maintained for prestige. Mothers reported that it is normally not acceptable to slaughter animals in their culture unless during certain special occasions such as circumcision and after birth. An instance is the case where the healthy animals especially the bulls with long horns are treasured so much so they cannot be slaughtered. They just grow old and die. This leads to inadequate intake of meat among pastoralists who are presumed to majorly consume meat among other animal products.

The respondents highlighted that Maasai do not sell animals or animal products and this limits income generation for the family. This places a challenge on the food sources to the household, as income would improve the food basket through purchases of other foods.

The Maasai culture prohibits the consumption of wild animals, chicken and fish which limit the food scope. This leads to food insecurity especially when there is none or minimal animal products. Among the Maasai, fish consumption is not common as they perceive the aquatic animals not fit for human consumption. Thus, fish being one of the good and easily available sources of omega 3 fatty acids is not consumed. Chicken is normally perceived to be a bird which should not be eaten. Green vegetables are particularly perceived as livestock feed rarely consumed. Intake of some vitamins and minerals mainly derived from vegetables is therefore low. Culturally, Maasai believe that they should not consume milk and meat during the same meal. According to the respondents, such an act is perceived as destroying the whole animal and is disrespect to the highly treasured animals. Since they depend on livestock products and the fact that they cannot eat them at the same time leads to inadequate nutrient consumption.

The Maasai believe that land is only for grazing and not cultivation. It was highlighted that utilizing the land for crop farming is a crime against nature. Once you cultivate the land, it is no longer suitable for grazing. This contributes to low crop production which consequently leads to low consumption of food crops. This has also led to a slow progression of efforts to promote agro-pastoralism. The land in the Maasai community is communally owned therefore any activity to be done on the land must be agreeable by the community. With overreliance on animal products and minimal crop production, the diets lack diversification.

Culture encourages the feeding of an infant below six months with blood, animal’s milk and bitter herbs. They also believe in feeding the baby with fatty concoction laced with ghee two weeks after birth. This reduces the rate of exclusive breast feeding. Without exclusive breastfeeding, infants are likely to have a low immune system becoming prone to infections. Other than being cheap and readily available, breast milk has been shown to have health benefits to both the mother and the child. Exclusive breastfeeding rates are low among the Maasai pastoralist [3].

In the Maasai community, fathers are given preferential treatment over the women and children. The men are served first and in large amounts while children and women are served last therefore poor intra-household food distribution. In case of inadequate food in the households, the children don’t get enough.

The Maasai consume raw meat, milk and blood. The main animal parts that are eaten raw are kidney, liver and the tail of a sheep. This practice is likely to lead to infections, worm infestation and other diseases like brucellosis.

The Maasai ferments milk as a common practice. This is mainly done in the event of excess milk for preservation with some herbs added to the milk for medicinal properties. Fermentation process improves bioavailability of micronutrients like iron and zinc and not only adds to the flavour of the milk but also improves the nutrient content milk [36]. Consumption of fermented milk has been shown to reduce the instances of gastrointestinal infections and is also beneficial in reduction of the levels of milk allergens common to fresh milk [29].

The Maasai live together in clusters called *Manyattas* adopt a socialism way of living. This promotes food security as well as food insecurity. During migration, families who move are housed by others in their destination for the time they will be in the area. Similarly, if a household has food they have responsibility to share with other households that do not have making food inadequate once shared. The belief in socialism ensures food sharing and availability.

They Maasai use traditional healing and intake of herbs as medicine and this has contributed to poor health seeking behaviour. Due to this, they miss on basic health services that are normally provided such as health and nutrition education. This is in agreement to a study by Bhui [8], which noted that, culture affected health practices. At two weeks after birth, the new born is given traditional herbs as this is perceived to prevent them from getting diseases. This has lead to low rates of immunization and vitamin A supplementation coverage [3].

## Conclusion

This study concludes that culture influence the dietary practices among children under five years. Since Maasai have a strong culture that affect dietary practices among children. The culture is associated with food insecurity due to lack of sale or slaughter of animals as well as low crop production. By avoiding some available foods, the amount of food available for consumption is narrowed. Early introduction of foods to children before six months limits the WHO exclusive breast feeding practices. The low level of education is also a contributing factor to poor dietary practices.

## Recommendations

This study recommends initiation of programs to create awareness on the influence of culture on dietary practices through behaviour change communication with a view to ensure that the effects of some cultural beliefs are addressed so as to improve health and nutritional status.
